# Overcoming the Open-Circuit Voltage Losses in Narrow Bandgap Perovskites for All-Perovskite Tandem Solar Cells

**DOI:** 10.1021/acsmaterialslett.4c01699

**Published:** 2024-10-23

**Authors:** Yekitwork Abebe Temitmie, Muhammad Irfan Haider, Daniele T. Cuzzupè, Lucia V. Mercaldo, Stefan Kraner, Paola Delli Veneri, Amare Benor, Azhar Fakharuddin, Lukas Schmidt-Mende

**Affiliations:** † Department of Physics, University of Konstanz, 78464 Konstanz, Germany; ‡ Department of Physics, University of Bahir Dar, 6000 Bahir Dar, Ethiopia; § Italian National Agency for New Technologies, Energy and Sustainable Economic Development (ENEA), Portici Research Center, 80055 Portici, Italy

## Abstract

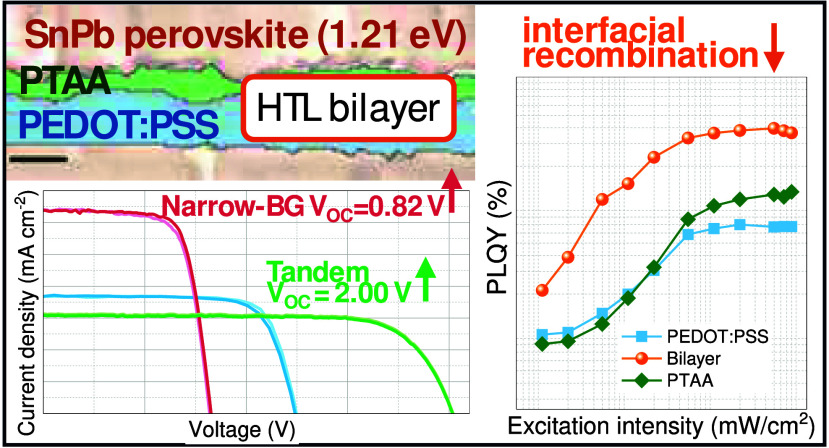

Narrow-bandgap (NBG) perovskite solar cells based on tin–lead mixed perovskite absorbers suffer from significant open-circuit voltage (*V*_OC_) losses due primarily to a high defect density and charge carrier recombination at the device interfaces. In this study, the *V*_OC_ losses in NBG perovskite single junction cells (*E*_g_ = 1.21 eV) are addressed. The optimized NBG subcell is then used to fabricate highly efficient all-perovskite tandem solar cells (TSCs). The improvement in the *V*_OC_ is achieved via the addition of a thin poly(triarylamine) interlayer between the poly(3,4-ethylenedioxythiophene) poly(styrenesulfonate) (PEDOT:PSS)-based hole transport layer (HTL) and the NBG perovskite. The optimal bilayer HTL results in a champion power conversion efficiency (PCE) of 20.3%, compared to 17.8% of the PEDOT:PSS-based control device. The *V*_OC_ improvement of the single-junction NBG cell is also successfully transferred to all-perovskite TSC, resulting in a high *V*_OC_ of 2.00 V and a PCE of 25.1%.

Performance improvement in solar cells is a subject of intense research as higher-performing cells reduce energy cost per square meter. The performance enhancement in different generations of solar cells has been achieved through the understanding of the device physics and optimizing different materials and their interfaces in their device stack. In recent years, organic–inorganic metal halide perovskites-based solar cells (PSCs)^1−3^ have achieved certified power conversion efficiency (PCE) exceeding 26%.^4,5^ Halide perovskites offer unique optoelectronic properties such as a long carrier diffusion length, high absorption coefficient, and suitable and tunable bandgaps.^6,7^ Despite the impressive PCEs, PSCs face numerous challenges that must be addressed prior to their commercialization such as their long-term operational stability.^8,9^ PSCs are typically made in a regular (n-i-p) or inverted (p-i-n) architecture, and herein, the choice of the electron and hole transport layers strongly determines the performance and stability of devices. For instance, the widely employed organic hole transport layer (HTL) 2,2′,7,7′2,2′,7,7′-Tetrakis[N,N-di(4-methoxyphenyl)amino]-9,9′9,9′ spirobifluorene (Spiro-MeOTAD) in n-i-p PSCs is readily prone to degradation upon exposure to humidity.^10^ The choice of HTLs strongly affects the photophysical properties of perovskite layer atop, as well as various electronic processes at the interfaces such as charge extraction, charge accumulation and the associated hysteresis in devices, recombination, and so on.^11−13^

An advantage offered by halide perovskite materials is the broad tunability of their optical bandgap in the range of 1.24 to 3.55 eV,^14^ which makes them particularly suitable for application in tandem solar cell (TSC) configuration.^15^ This can be achieved via compositional engineering, for instance, substituting the central element lead (Pb) with tin (Sn) or halide substitution at the X-site. Sn-containing perovskites offer narrower bandgaps than their Pb-counterparts, which are desirable for bottom cell in all-perovskite TSCs, the optimum being the bottom cell bandgap to be ∼1.2 eV and the top cell bandgap to be ∼1.7 eV.^16−18^ Pure Sn perovskites particularly suffer from stability issues due to the oxidation of Sn^2+^ to Sn^4+^.^19^

The major advantage of mixed tin–lead halide perovskites lies in the particularly narrow bandgaps (NBGs) that are achieved due to the bandgap bowing effect,^20^ resulting in energy gaps smaller than both the neat-Sn and the neat-Pb counterparts. For Sn–Pb perovskites, the achievable bandgaps lie between 1.2 and 1.4 eV.^21,22^ Furthermore, the copresence of tin and lead in the lattice improves the atmospheric stability of the material. However, Sn–Pb-based NBG perovskites show low open-circuit voltage (*V*_OC_) in devices, which directly limits the device performance.^23,24^

The low *V*_OC_ is due to the nonoptimal interfaces leading to additional recombination losses in the device as well as due to a lower absorber quality of Sn-based perovskite.^25−29^ It has been reported that the *V*_OC_ of the Sn-based PSCs is low due to the heavy p-type doping of the materials, attributed to the oxidation of Sn^2+^ to Sn^4+^, which acts as a p-type dopant in the structure, resulting in excessive dark-carrier concentration and high photocarrier recombination.^30,31^

To date, most highly efficient Sn–Pb mixed PSCs employ an organic HTL based on poly(3,4-ethylenedioxythiophene) poly(styrene sulfonate) (PEDOT:PSS),^32,33^ which is among the key reasons for the inferior *V*_OC_. Yang et al. introduced Br^–^ into the mixed Sn–Pb MAPb_0.5_Sn_0.5_(I_0.8_Br_0.2_)_3_ perovskite and demonstrated a PCE of 17.63% with a small *V*_OC_ deficit of 0.45 V.^34^ In another work, the *V*_OC_ of the devices was improved by 70 mV by employing mixed precursor solution consisting of formamidinium tin iodide (FASnI_3_) and methylammonium lead iodide (MAPbI_3_) and a surface passivation strategy using PEABr (PSCs yielding a PCE of 15.15%).^35^ Replacing the acidic PEDOT:PSS by low-temperature processed NiOx resulted in a PCE of 18.77%, and the PSCs showed enhanced stability, retaining 96% of the initial PCE after 500 days of shelf life storage.^36^

In this work, we selected polytriarylamine (PTAA) as an interlayer between the PEDOT:PSS and the (FASnI_3_)_0.6_(MAPbI_3_)_0.4_ perovskite (henceforth termed as “NBG perovskite”) to overcome the *V*_OC_ losses typical of these perovskites. We compared three different HTL strategies: PEDOT:PSS-based PSCs, PTAA-based PSCs, and cells employing PTAA/PEDOT:PSS bilayer HTL. Strikingly, the devices employing the bilayers delivered a PCE of 20.3%, up from 17.8% for PEDOT:PSS-based PSCs and 16.0% for PTAA-based single-junction PSCs. The bilayer HTL-based PSCs showed a mean *V*_OC_ of 0.80 V, an absolute 50 mV enhancement from the PEDOT:PSS based counterparts. The enhanced PCE and *V*_OC_ of the bilayer-based PSCs are attributed to improved charge extraction and reduced interfacial recombination. We further demonstrate that the optimized NBG device employing the highly efficient bilayer as HTL can serve as the bottom subcell in an all-perovskite TSC. By combining the optimized NBG perovskite subcell with the wide bandgap (WBG) composition (Cs_0.3_FA_0.6_MA_0.1_Pb(I_0.7_Br_0.3_)_3_ (hereinafter “WBG perovskite”)), we demonstrate a remarkable champion *V*_OC_ of 2.00 V and a high PCE of 25.1%.

## Screening Hole Transport Layers for High-Efficiency PSCs

**Figure 1:**
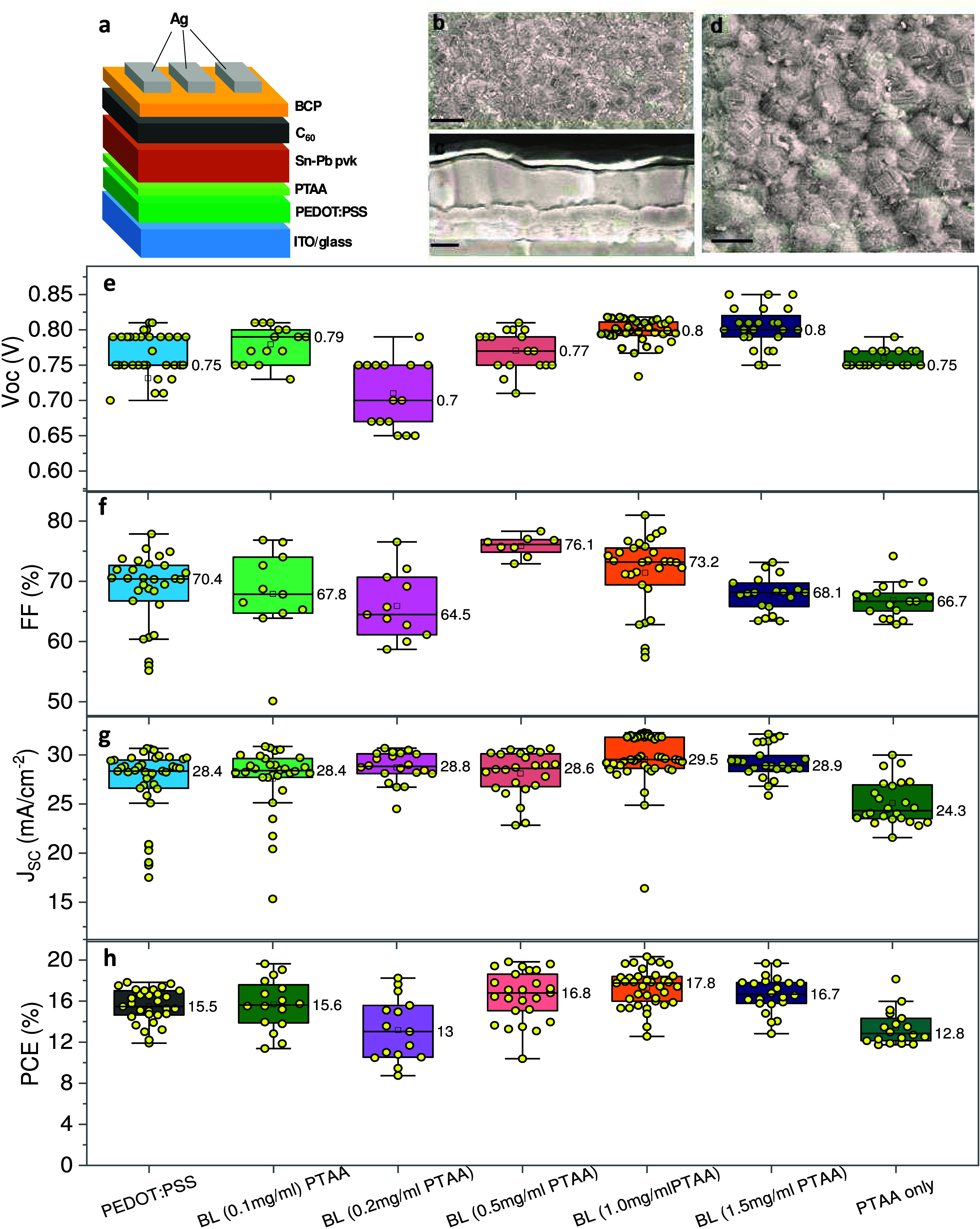
(a) Device architecture of the NBG Sn–Pb PSC used in this work. (b, d) Surface morphology of the perovskite deposited atop bilayer HTL (scale bars 1 μm and 400 nm, respectively). (c) Cross-sectional SEM image of complete PSC stack (scale bar 200 nm). (e–h) Box chart representing *V*_OC_, FF, JSC, and PCE of PSCs employing various HTLs. The data refer to several devices from at least five different batches fabricated in this work. The HTL series 0.1–1.5 mg/mL PTAA refers to the concentration of the HTL precursor solution. The PTAA layers were cast on top of the PEDOT:PSS layer to make bilayers (BL). The concentration of the PTAA-only case is 1.0 mg/mL. The data labels in (e–h) show mean values.

Figure 1a illustrates the typical architecture of a single-junction PSC employing an NBG Sn–Pb perovskite. Figure 1(b-d) confirms that a compact and pinhole-free perovskite film is obtained, which is a crucial prerequisite for achieving a high device performance. Initially, a series of HTLs were screened to identify the most effective HTL combination for these devices. The selection criteria included energy-level alignment with the NBG perovskite, as well as considering the best-performing HTLs from their Pb-based counterparts. These HTLs included PEDOT:PSS and PTAA chosen based on their possible energy-level alignment with NBG perovskite as well as based on their track record in Pb-PSCs.^37,38^ The energy level alignment between the HTL and the perovskite is essential to preventing charge recombination losses. The HTL’s valence band edge should be higher than the perovskite’s valence band edge to allow for efficient hole transfer, while its valence band edge should be lower than the cathode’s conduction band edge to ensure complete extraction of holes.^21^ The ionization energy values of HTLs and NBG perovskite given in the Supporting Information (Figure S1) confirmed a better energy alignment for bilayer and NBG perovskite, assuring a better charge extraction. The PTAA concentrations considered were 0.1, 0.2, 0.5, 1.0, and 1.5 mg mL^–1^, and the champion *V*_OC_ values obtained for each concentration were 0.81 0.79, 0.81, 0.82, and 0.83 V, respectively. While a concentration of 1.5 mg mL^–1^ demonstrated the highest *V*_OC_ among the five concentrations employed in this work, the optimal concentration for achieving a trade-off between *V*_OC_, FF, and overall PCE was found to be 1.0 mg mL^–1^.

Based on the results shown in Figure 1(e-h), we chose three HTLs for further investigations, namely, PEDOT:PSS-only, PTAA-only (1 mg mL^–1^), and the bilayer HTL employing the PTAA film (concentration of 1 mg mL^–1^) atop PEDOT:PSS. The chemical structures of these HTL materials are displayed in Figure S2.

**Figure 2:**
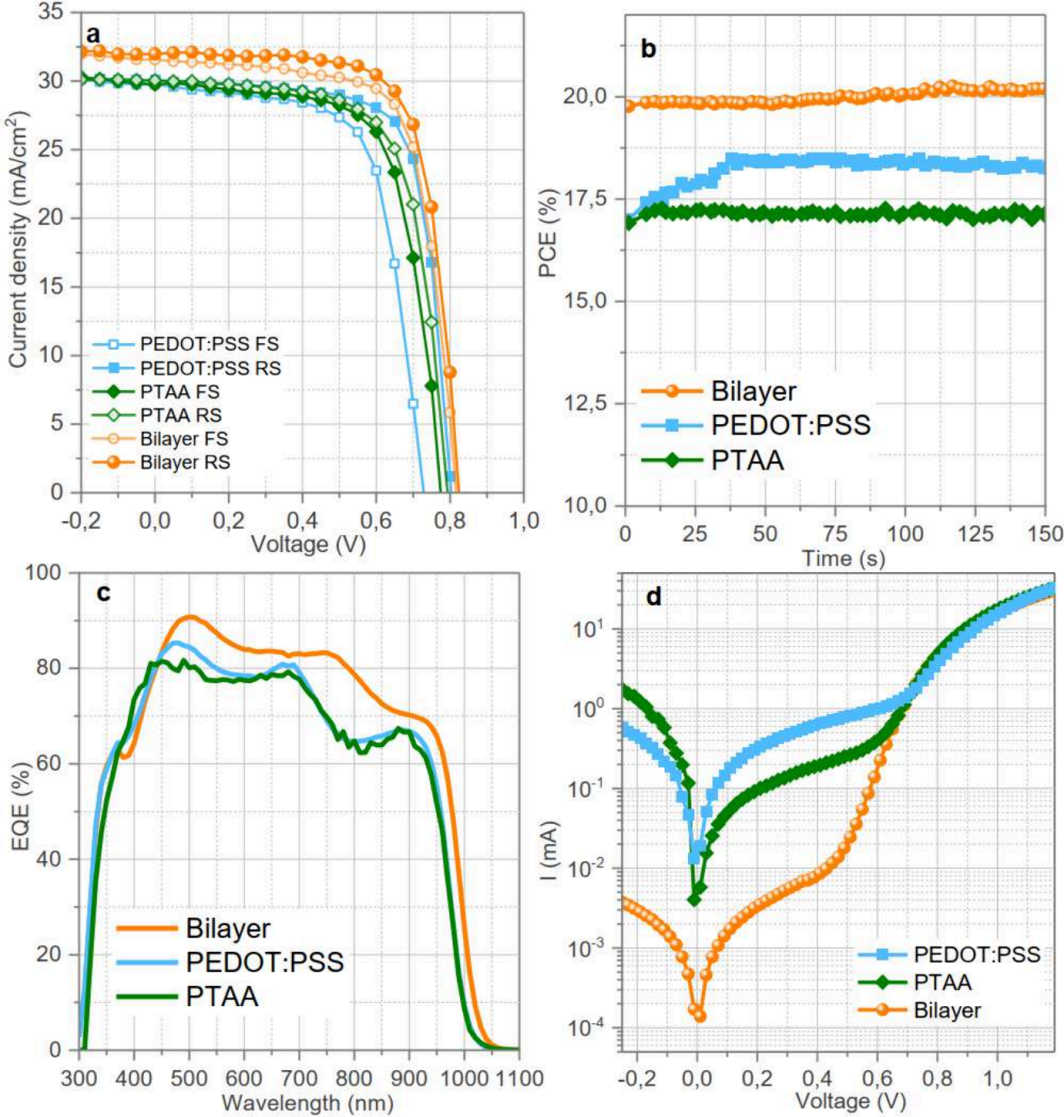
(a) Current–voltage (*J*-*V*) curves, (b) maximum power point tracking, (c) external quantum efficiency, and (d) dark *J*-*V* curves of PSCs employing all three HTLs (with an antireflection coating (ARC, MgF_2_, 70 nm).

By comparing three HTLs from Figure 1(e-h), the bilayer approach showed the most efficient device, which exhibited a PCE of 20.3%, *J*_SC_ of 32.2 mA cm^–2^, *V*_OC_ of 0.82 V, and FF of 81.0%. The champion PEDOT–PSS-based device resulted in a PCE of 17.8%, *J*_SC_ of 30.6 mA cm^–2^, *V*_OC_ of 0.81 V, and FF of 78.0%. The champion PTAA-only based device showed an efficiency of 16.0%, a *J*_SC_ of 30.0 mA cm^–2^, a *V*_OC_ of 0.79 V, and an FF of 69.9% (Figure 2a). The bilayer HTL configuration also shows reduced hysteresis, suggesting balanced charge extraction and improved charge transport compared to the PTAA- and PEDOT:PSS-based counterparts (Table S1). PCE obtained from maximum power point tracking (MPPT) (Figure 2b) shows an initial rise in the PCE upon light soaking for PEDOT:PSS or PTAA-HTLs, whereas the bilayer structure shows a stable PCE for 150 s.

To confirm the improved *J*_SC_ in the bilayer HTL, external quantum efficiency (EQE) measurements of representative PSCs were performed (Figure 2c). Quantitatively, *J*_SC,EQE_ (calculated by *J*_SC, EQE_ = e ∫EQE (λ) *S* (λ) dλ, where *S*(λ) is the AM1.5G sun spectrum in units of photons per second) are 29.4, 27.8, and 27.4 mA cm^–2^ for bilayer, PEDOT:PSS, and PTAA, respectively. These values are in agreement with the *J*_SC_ measured by the *J*-*V* curves, considering that EQE and *J*-*V* measurements were performed on devices without and with an antireflection (AR) coating, respectively, and show the same relative increase of the current when replacing the single HTL with the double HTL. The influence of AR coating and its optimal thickness on the *J*_SC_ is described in Figure S3 and Figure S4. The higher EQE in the full spectral range for the devices with bilayer HTL is indicative of an improved charge collection with respect to the individual HTL counterparts.

The origin of reduced recombination and hence the improved *V*_OC_ and FF can be understood by comparing the dark *J*-*V* curves in Figure 2d, which shows a notable difference in the leakage current values among the three different HTLs. The bilayer-HTL PSCs exhibited nearly 15 times suppressed leakage current compared to devices utilizing PEDOT:PSS and PTAA as individual HTLs. For the bilayer-based PSCs, the introduction of the PTAA interlayer improves the perovskite film coverage and reduced the pinhole formation. The bilayer structure contributed to better interface quality, reducing defects and minimizing pathways for leakage currents. A lower leakage current in the case of bilayer HTL is attributed to a complete coverage of HTL over the ITO substrate, which ensures opposite charge carriers from the perovskite layer do not recombine with the holes in the HTL, thus contributing to the higher *V*_OC_ and higher FF in the case of the bilayer HTL.

## Interface-Dependent Photophysical Effects

**Figure 3:**
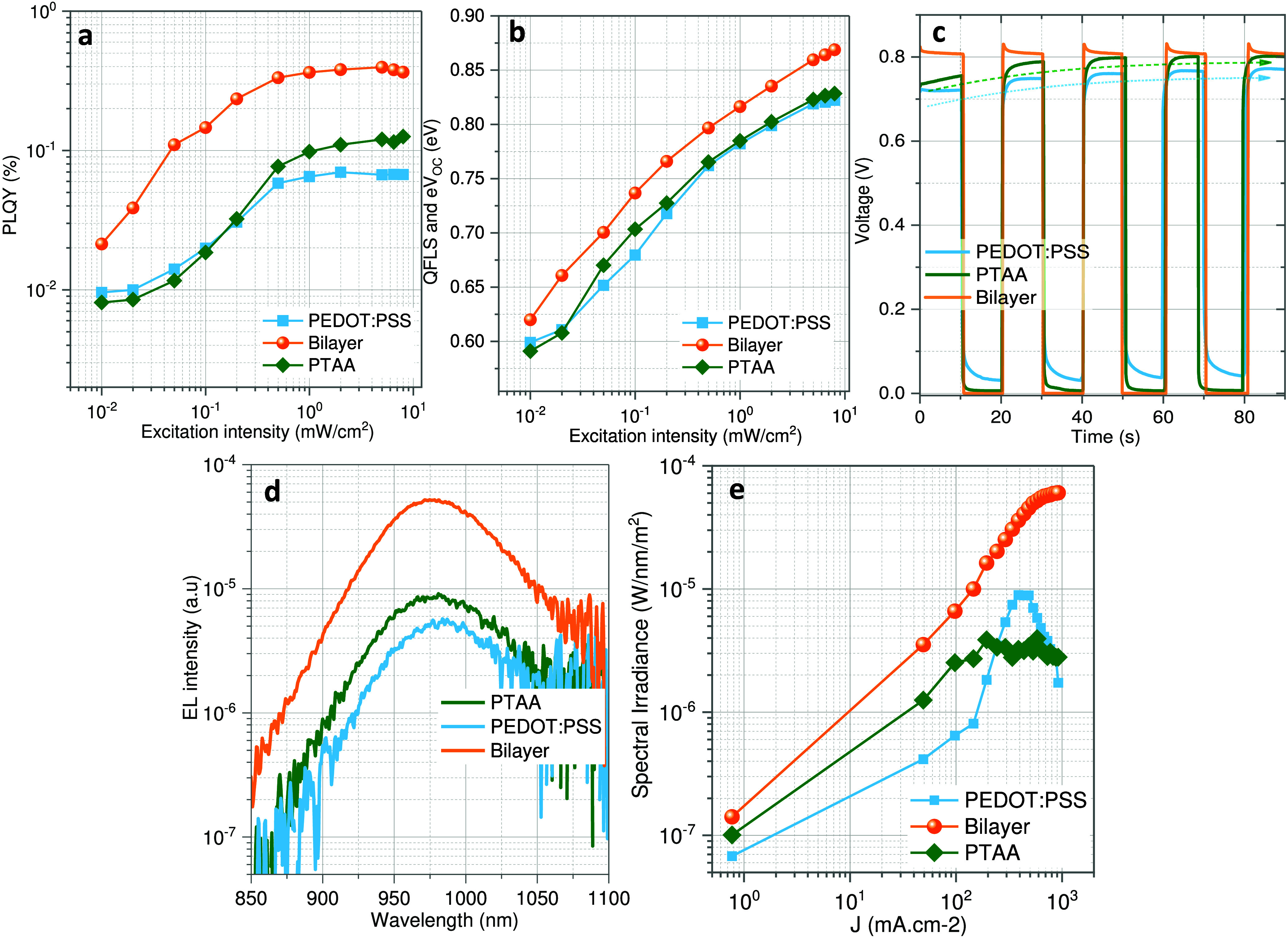
(a) and (b) show the photoluminescence (PL) quantum yield and QFLS of perovskite films deposited atop three different HTLs. (c) shows the rise and decay of the *V*_OC_ of the PSCs employing the three HTLs, measured at 1-sun conditions. (d) Electroluminescence (EL) curves of three representative PSCs at an injection current density of 50 mA/cm^2^, measured in air. (e) EL intensity as a function of injected current density for all three PSCs.

We attribute the different performance of the three HTLs primarily to the fact that the properties of the perovskite films are, as is well-known, substrate-dependent. The surface topography of perovskite films on the selected HTLs (Figure S5) established a better smoothness in case of bilayer with smaller root-mean-square (RMS) value of (37.68 nm) in comparison to PEDOT (68.32 nm) and PTAA only (44.36 nm) highlighting further toward the improved properties of perovskite film. Even if in the case of the bilayer and PTAA only the underlying layer is nominally the same (PTAA), we note how the RMS value is different, highlighting that the production of the PTAA layer by spin coating is affected by the underlying substrate layer. It can be concluded that the fabrication of a PTAA layer on top of PEDOT:PSS-coated substrates results in more homogeneous and high-quality layers as compared to the same process on bare ITO-coated glass substrates, ultimately influencing the interface with the perovskite layer and improving the PSC performance. This is confirmed by photoluminescence quantum yield (PLQY) measurements, suggesting a better interface quality of the bilayer-HTL/perovskite stack, with around 3-fold higher PLQY noted for bilayer HTL than the other two HTLs (Figure 3a-b). This also correlates to a higher quasi-Fermi level of splitting (QFLS), also referred to as implied *V*_OC_.^39^

We further recorded the rise and drop in the *V*_OC_ of the PSCs at 1-sun conditions employing three HTLs (Figure 3c). While the PEDOT:PSS and PTAA-based HTLs show a slow rise in the *V*_OC_ over a time span of several seconds, which might be related to capacitive effects in the device. The bilayer HTL-based PSC quickly reaches its maximum *V*_OC_. In an ideal case, the device should reach its maximum *V*_OC_ and should not show a temporal dependence upon light soaking, a trend clearly more pronounced in the bilayer HTL PSCs hinting at improved interfacial properties with Sn–Pb perovskite.

To confirm the improvement in the PSCs in the bilayer HTL, we recorded the electroluminescence (EL) spectra of the PSCs. Figure 3d,e shows EL spectra and EL yields as a function of injection current densities for all three PSCs. The EL measurements at a given current density exclude the resistive effects that might be present in the devices due to different energy level alignments or due to the different charge transport characteristics of the HTLs.^40^ The EL spectra at a given current density of 50 mA cm^–2^ show the highest EL for bilayer HTL, followed by PTAA and PEDOT:PSS. This is a direct confirmation of higher radiative recombination in the bilayer HTL-based PSCs, which originates from the improved interfacial properties at the HTL/NBG perovskite interface. The trends in the EL intensity validate the *V*_OC_ values in the cells. It further suggests a higher EL in the low injection current density, indicative of a significantly lower trap density in these films.^41^ In general, PTAA-based HTLs show a saturation of the EL intensity at an elevated injection current density, while the EL of the PEDOT:PSS-based film shows a drop.

The higher quality of perovskite films on PTAA HTLs is confirmed by time-resolved PL (TRPL) transients (Figure S6a,b). The PTAA-only HTL shows the highest carrier lifetime, followed by the bilayer HTL. However, a higher carrier lifetime does not always lead to higher PCE. A rapid PL decay (or PL quenching) can indicate both efficient charge extraction and high interfacial or bulk defect density, the former being desirable for higher PSC performance and the latter being undesirable and leading to lower performance. We attribute the lower PCE of PTAA-based devices despite the longer carrier lifetime to poor charge extraction, likely due to poor conductivity. On the other hand, the rapid PL decay of the films grown on PEDOT:PSS combined with the lower efficiency of the PEDOT:PSS-based devices hints toward increased interfacial trapping.^42^ The bilayer HTL balances increased radiative recombination and charge extraction, resulting in higher *V*_OC_ and FF. The detailed fitting parameters are presented in Table S2. Comparing front- and back-side PL provides further insights into the charge extraction and film quality. Stronger front-side PL suggests an inefficient charge extraction.

**Figure 4:**
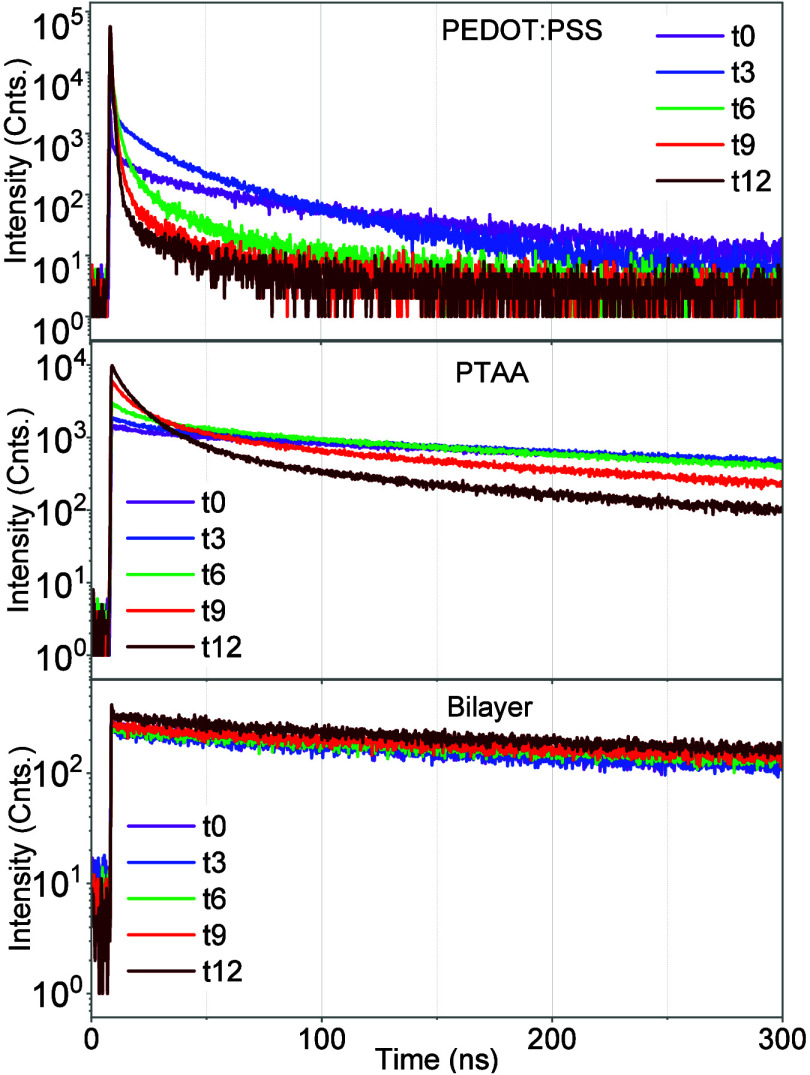
PL transients of the ITO/HTLs/perovskite excited from the glass side, measured in air under continuous laser irradiation (pulsed laser, λ_exc_ 450 nm, repetition rate 200 kHz). The PL transients were measured periodically every 3 min (*t*_0_... until *t*_12_).

In order to track whether the different HTLs lead to different ambient/photostability, we tracked temporal PL in air under continuous laser irradiation for several minutes. TRPL does not show any notable changes for the bilayer, followed by PTAA, whereas the PEDOT:PSS HTL-based perovskite film underwent the most pronounced changes (Figure 4). This higher initial drop in the PL intensity for the PEDOT:PSS HTL might be due to a higher trapping at the PEDOT:PSS/perovskite interface that leads to a drop in the TRPL lifetime. Generally, the performance in the perovskite-based devices is attributed to their longer carrier lifetime, and often superior quality films are coupled with slower PL decays.^43^ TRPL lifetime for PTAA only based perovskite films revealed a slow PL decay for the initial measurements, followed by a reasonable drop after 6 min of photoirradiation, hinting that the continuous light exposure leads to creation of defect states. The bilayer HTL-based perovskite films exhibit consistent TRPL under continuous laser irradiation even for a prolonged exposure, arguing the high quality of bilayer/perovskite interface. These results are also consistent with the PLQY and EL findings and are complemented by the device performance.

## All-Perovskite Tandem Solar Cells

**Figure 5:**
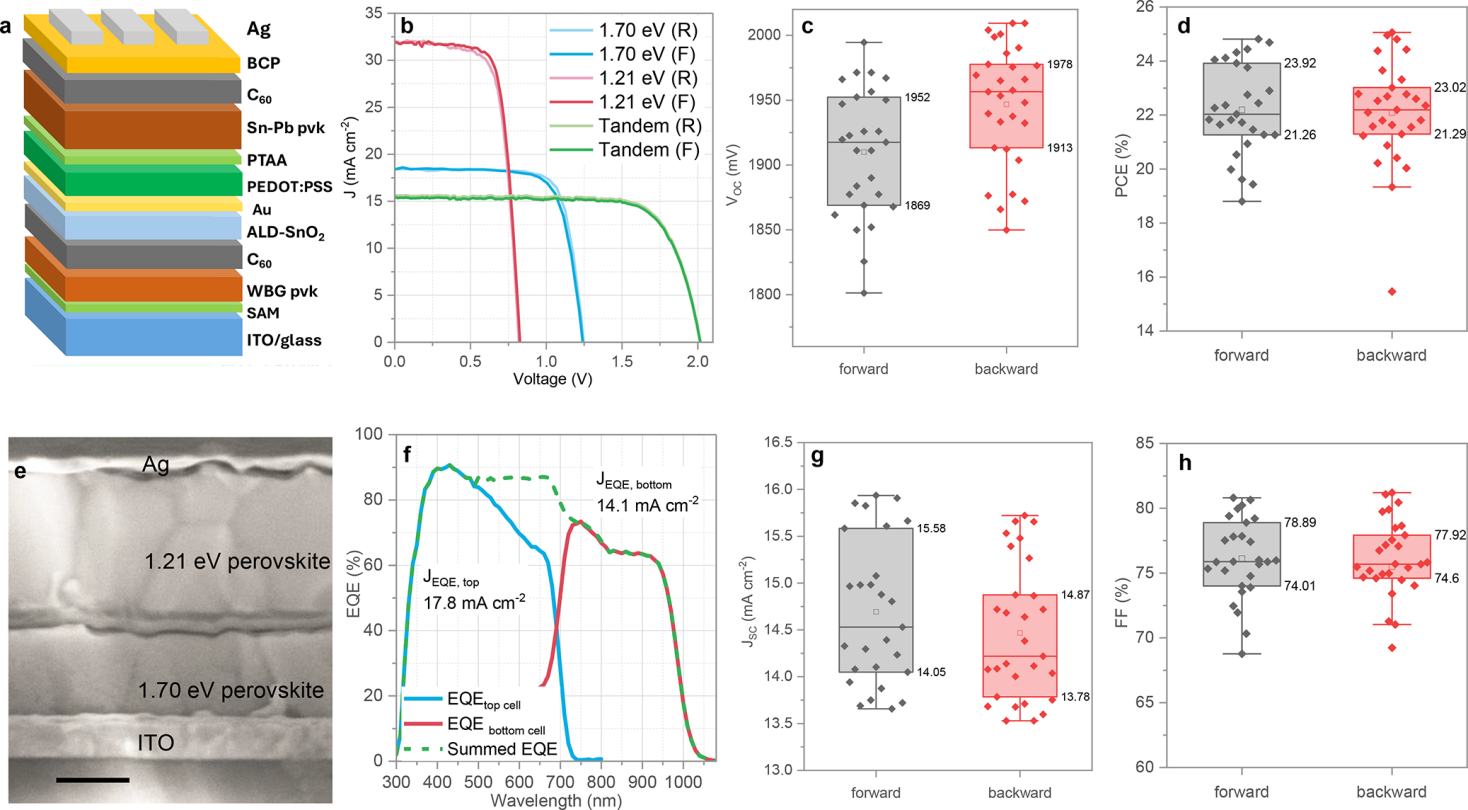
(a) Device architecture of all-perovskite TSCs. (b) *J*-*V* curves of corresponding single-junction PSCs and all-perovskite TSC measured inside a N_2_-filled glovebox. (c, d, g, and h) show statistical analysis of 28 all-perovskite TSCs. The box labels show mean, the highest, and the lowest values from all devices. (e) Cross-sectional view of all-perovskite TSC employing a bilayer HTL (Scale bar 250 nm). (f) EQE spectra of all-perovskite TSC measured in air.

The gains made in the single-junction NBG cells are applied to fabricate an all-perovskite TSC that also involves a separately optimized WBG perovskite (*E*_g_ = ∼1.70 eV, Cs_0.3_FA_0.6_MA_0.1_Pb(I_0.7_Br_0.3_)_3_) as the top cell and the optimized NBG perovskite (*E*_g_ = 1.21 eV) as the bottom cell. Both the WBG and the NBG perovskite layers were processed in a N_2_-filled glovebox (with the exception of PEDOT:PSS as HTL as well as the recombination layer and atomic layer deposition of SnO_*x*_ (see experimental section in the Supporting Information). The configuration of all-perovskite TSCs presented in Figure 5a is as follows: ITO/SAMs/WBG/C_60_/ALD-SnO_*x*_/Au/bilayer (PEDOT:PSS/PTAA)/NBG/C_60_/BCP/Ag.

Figure 5b displays the *J*-*V* curves of the best performing bilayer-based NBG bottom cell and all-perovskite TSCs with a minimal hysteresis, demonstrating a PCE of 20.3% and 25.1%, *V*_OC_ of 0.82 and 2.00 V, *J*_SC_ of 32.3 mA cm^–2^ and 15.9 mA cm^–2^, and FF of 81.0% and 81.2%, respectively. The high PCE of tandem devices, with a remarkable *V*_OC_ of 2.00 V, is obtained by utilizing the bilayer HTL discussed in the previous sections for the NBG bottom cell (Figure 2a), which, as we have seen, contributes to enhanced photovoltaic (PV) parameters. Additionally, this value is made possible by the optimization of the WBG top cell, where the gas-quenching method was employed for the perovskite formation. Figure 5c,d,g,h compares the PV parameter for the reverse and forward scans of corresponding 28 tandem devices obtained from several identical runs. Here, the results with narrow distributions indicate good reproducibility and minimal hysteresis for tandem devices. The improved photovoltaic parameters of tandem devices can also be linked to the crack-free, compact, and high-quality perovskite films (absorber layers) for both the WBG and NBG subcells as demonstrated in the cross-sectional SEM of all-perovskite TSCs (Figure 5e). To reduce the mismatching of the *J*_SC_ of the top and bottom subcells, the thicknesses of WBG and NBG absorber layers were optimized to be 400 and 700 nm, respectively. In Figure 5f, the EQE spectra are presented for both the top and bottom subcells along with the summed EQE for a representative tandem device. The integrated current density of top WBG and bottom NBG subcells from EQE spectra are found to be 17.8 and 14.1 mA cm^–2^, in agreement with the mean *J*_SC_ values in the *J*-*V* measurement data set and providing evidence that the device is limited by the NBG component cell.

## Supporting Information Available

Materials and methods, energy level alignment obtained by PESA, chemical structures of the HTLs, additional J-V results, AFM images, TRPL plots and fitting parameters (PDF)
