# Supplementary material for: Overcoming the Open-Circuit Voltage Losses in Narrow Bandgap Perovskites for All-Perovskite Tandem Solar Cells
Source: ACS Mater Lett. 2024 Oct 23;6(11):5190–8. doi: 10.1021/acsmaterialslett.4c01699 (PMC11539106; doi:10.1021/acsmaterialslett.4c01699)
Supplement: Supplementary file 1 — tz4c01699_si_001.pdf [file tz4c01699_si_001.pdf]

Supporting Information

Overcoming the open circuit voltage losses in narrow bandgap perovskites for all-perovskite tandem solar cells

Yekitwork Abebe Temitmie[1,2], Muhammad Irfan Haider[1], Daniele T. Cuzzupè[1], Lucia V. Mercaldo[3], Stefan Kraner[1], Paola Delli Veneri[3], Amare Benor[2], Azhar Fakharuddin[*,1], Lukas Schmidt-Mende[*,1]

Materials and methods.

***Materials**:* ITO-coated glass slides (15Ω/sq) were purchased from *Lumtec Inc.* PEDOT:PSS water dispersion under the commercial name of *Clevios PVP Al 4083* was purchased from *Heraeus.* Lead (II) thiocyanate ($Pb(SCN)_2$, 99.5%), tin (II) fluoride (SnF_2), magnesium fluoride (MgF_2, 99.9%), were purchased from *Sigma Aldrich.* Methylammonium iodide (MAI) and formamidinium iodide (FAI) were purchased from *Greatcell Solar*. Tin (II) iodide (SnI_2), 99.999% was purchased from *Thermo Scientific*. Lead (II) iodide (PbI_2, 99.99%) was purchased from *TCI Chemicals.* Poly[bis(4-phenyl) (2,4,6-trimethylphenyl) amine] (PTAA), buckminsterfullerene (C_{60}), bathocuproine (BCP), silver (Ag), dimethylformamide (DMF, anhydrous 99.8%), dimethyl sulfoxide (DMSO, >99.9%), toluene (anhydrous, 99.8%), and diethyl ether (anhydrous) were purchased from *Across Organics.*

***Preparation of NBG perovskite**:* The Sn-Pb perovskite with composition $(FASnI_3)_{0.6}(MAPbI_3)_{0.4}$ was prepared by mixing $FASnI_3$ and $MAPbI_3$ solutions with a volume ratio of 0.6:0.4 as described elsewhere [53]. For the $FASnI_3$ solution with a concentration of 1.15 M, 18 mg of SnF_2 (10 mol%), 197.8 mg of FAI, and 428.4 mg of SnI_2 were dissolved in an 800 μL DMF and 200 μL DMSO mixture and stirred overnight at room temperature. For the $MAPbI_3$ solution with a concentration of 1.15 M, 530 mg of PbI_2, 183 mg of MAI, and 3.5 mol% $Pb(SCN)_2$ additive were dissolved in 630 μL DMF and 70 μL DMSO, and the mixture was stirred for 30 minutes at 70°C and then stirred overnight at room temperature. Subsequently, the $FASnI_3$ precursor solution was filtered through a 0.2 μm PTFE filter, then the two precursors were combined using a 0.6:0.4 $FASnI_3$:$MAPbI_3$ ratio. The resulting Sn-Pb precursor solution was stirred at room temperature for 30 minutes before use.

Fabrication of NBG perovskite solar cells: The ITO substrates were cleaned for 30 min using detergent, deionized water, acetone, and IPA under ultra-sonication, in this order. After being dried by nitrogen flow, the substrates were treated with UV-Ozone for 20 min. For the PEDOT:PSS deposition, the purchased dispersion was filtered through a 0.45 μm PVDF filter. The dispersion was deposited onto the cleaned ITO substrates by spin coating at 8000 rpm for 40 s, followed by annealing at 120°C for 15 min. After the deposition and annealing under ambient air, the substrates were quickly transferred into the glovebox. For PTAA-only devices, the PTAA solution was spin coated onto the cleaned ITO substrates at 8000 rpm for 40 s, followed by annealing 100°C for 3 min. For the bilayer-based devices, a PTAA thin film layer was spin coated on the top of PEDOT:PSS (bilayer) at 8000 rpm for 40 s and then annealed at 100°C for 3 min. Before perovskite deposition, the device stacks featuring PTAA on top (either PTAA only or bilayer) were treated with O_2-plasma for 2.5 sec to mitigate the poor wettability of PTAA. The perovskite precursors were spin-coated onto the HTLs at 4000 rpm for 60 s. Diethyl ether was used to initiate the perovskite crystallization, and it was dripped on the film during the spin-coating step 15 s after the beginning of the spinning. All perovskite films were annealed at 100°C for 10 min and then transferred into the next glovebox for thermal evaporation. Finally, 25 nm

C_{60}, 7 nm BCP and 100 nm Ag were sequentially evaporated on the top of perovskite films. An optimized 70 nm thick MgF_2 layer was thermally evaporated on the glass side of PSCs (at a rate of 1Å/s) as an anti reflection coating (ARC).

Fabrication of WBG perovskite solar cells: WBG perovskite solution (1M or 0.9 M) was made by mixing the following precursors in DMF:DMSO (3:1) at around 40°C in glovebox: $PbCl_2$ (0.02 M), CsI (0.3 M), MAI (0.1 M), FAI (0.6 M), $PbBr_2$ (0.45 M), and PbI_2 (0.55 M). The solution was completely dissolved and used without any filtering. The WBG PSCs were made on pre-cleaned ITO glass (same as NBG) and an 0.5 mM equimolar (50:50) mixture of self-assembled monolayers (MeO-2PACz and 2PACz) in ethanol was spin coated on ITO substrates in glovebox (3000 rpm for 30 s), followed by annealing at 100°C for 10 min. The perovskite solution (45 µL) was dropped onto a static cooled ITO substrate and the perovskite crystallization took place in the following steps. A N_2 flushing gun (pressure around 3.5 bar) was placed 2-3 cm above the spinning substrate; N_2 flushing started after 20 s of spinning (after perovskite dropping perovskite solution) and the substrates stayed under N_2 flushing for 15-20 s, until the films turned shinny and dark brown in color. For performance improvement, PEAI (1 or 2 mM) in CB:IPA (9:1) was spin coated (on spinning substrates) at 5000 rpm for 30 s (no further annealing was carried out). The device stack was completed by evaporating 25 nm of C_{60} (18 nm for tandem PSCs), 6 nm of BCP and 100 nm of Ag were sequentially evaporated to finish the device.

Fabrication of all perovskites tandem solar cell*:* For tandem PSCs, the device fabrication procedure is the same as WBG PSCs until C_{60} deposition (18 nm instead of 25 nm for single junction WBG PSCs). After the deposition of C_{60}, the perovskite films were transferred to an ALD system (Anric AT-410) to deposit SnO_2 in ambient conditions. As a precursor, tetrakis(dimethylamino)tin (IV) (TDMASn) was used at a temperature of 70°C, while water was used as an oxidant. The chamber temperature was set to 70°C. During exposure, a purge flow of 10 sccm was used, while each exposure step was 1 s. One ALD cycle consisted of 3 pulses of precursor, 12 s N_2 purge, 2 pulses of oxidant, 15 s N_2 purge. Here, 155 cycles were performed, resulting in thin films with a thickness of 20 nm. The substrates were transferred to a glovebox to deposit Au (0.5 or 0.6 nm) with an edge covered shadow mask (to avoid shunting between top and bottom cells). The cells were taken out to deposit HTL (PEDOT:PSS) to build a NBG cell atop. The same procedure of NBG cell fabrication (stated above) is followed to complete tandem devices.

Film and device characterizations*:* Photoluminescence quantum yield (PLQY) was measured using a *LuQY Pro* setup from *Quantum Yield Berlin*. Absorption spectra were recorded by a *Cary 5000* UV-vis-NIR spectrometer from *PerkinElmer*. Scanning electron microscope (SEM) images were acquired with a *Gemini 500 FESEM* system from *Zeiss* equipped with an in-lens detector. Time-resolved photoluminescence (TRPL) spectroscopy was performed with a *FluoTime 300* setup from *PicoQuant*. Kelvin probe force microscopy (KPFM) and atomic force microscope (AFM) were performed in a *NX10*

setup from *Park Systems.* Electroluminescence (EL) spectra were measured in a *Phelos* setup from *Fluxim*. The J-V curves of the PSCs were measured under simulated solar illumination at 100 mW cm^{-2}, AM 1.5G equivalent sunlight in a nitrogen-filled glovebox using an assembly made up of a *Keithley* 2400 source-meter unit, a *LOT* 300 W Xenon solar simulator, calibrated with a KG5-filtered Si reference diode (for WBG cells measurement) certified by *Fraunhofer ISE*. Space-charge limited current (SCLC) measurements were performed by measuring dark J-V curves of hole-only devices with configuration of (ITO/bilayer/NBG/P3HT/Ag). The external quantum efficiency (EQE) was measured with a Bentham PVE300 setup, by using Arkeo IPCE with tunable (300-1800 nm) light source without light bias and equipped with a 300 W Ozone Free Xe in AC mode having a chopper frequency at 16 Hz. Certified Si (Hamamatsu S1337) and Ge photodiodes were employed for the calibration of the monochromatic light intensity. For the tandem solar cells, the spectra of the two component cells were obtained by applying filtered bias light: red light to saturate the bottom cell and measure the top cell, blue light to saturate the top cell and measure the bottom cell.

Further characterization.

The ionization energy values obtained for the materials investigated in our study are presented in Figure S1. PEDOT:PSS exhibits 5.2 eV, consistent with its typical range. The bilayer interface has an energy of approximately 5.06 eV, reflecting the combined properties of both layers. In comparison to other HTLs, the bilayer seemed to be appropriate for efficient charge extraction, owing to well energetic alignment with that of NBG perovskite.

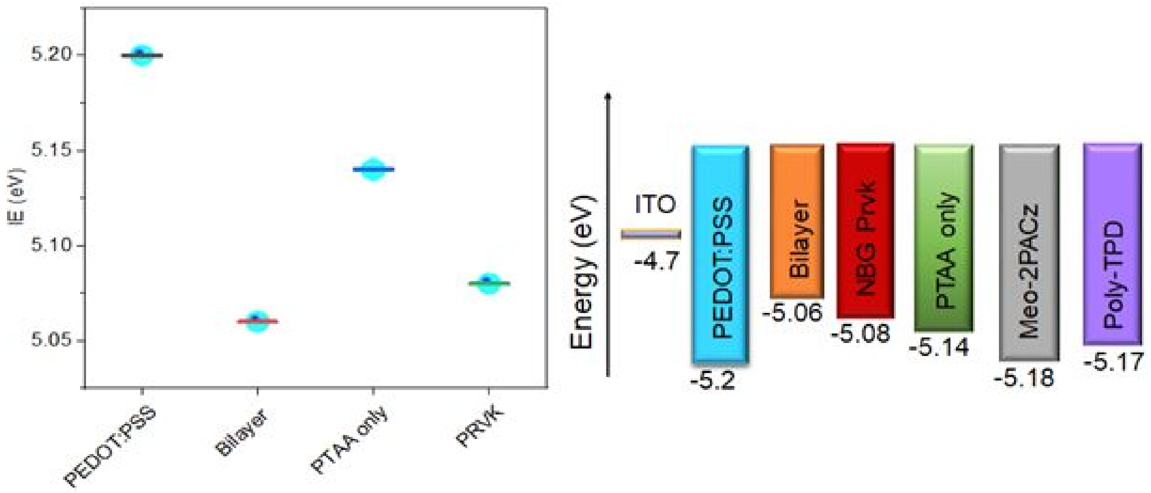

Figure S1. Ionization energy values of HTLs and NBG perovskite obtained by Photoelectron Spectroscopy in Air (PESA) and energy level alignment.

Hole transporting layers (HTLs) play a crucial role in perovskite solar cells (PSCs). The choice of the HTL significantly impacts PSC efficiency and stability. **Figure S2** shows

the chemical structures of the four HTLs, including PTAA and PEDOT: PSS that are used for performance comparison of PSCs. Those HTLs are critical components of inverted p-i-n PSCs, where alongside hole extraction and transport, they play an important role in surface passivation, perovskite crystallization, device stability, and cost management.

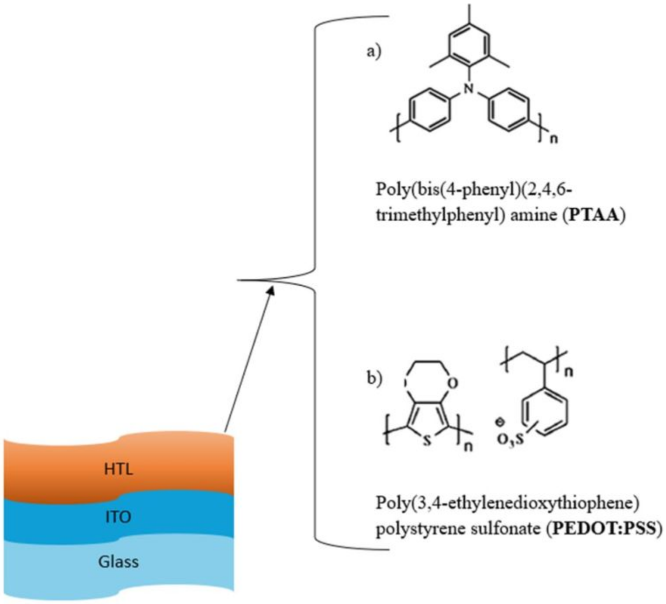

Figure S2. Chemical structures of the hole-transport layers in NBG PSCs: PTAA & PEDO:PSS.

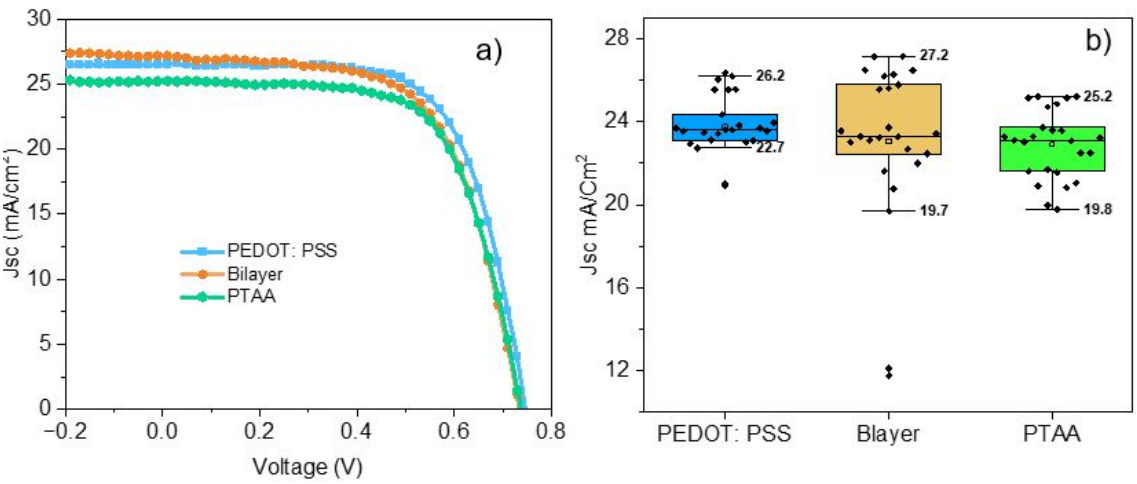

Figure S3. a) J-V curves and b) short circuit current of the three selected HTLs based PSCs without anti-reflection coating.

The presence of an anti-reflection coating (ARC) such as MgF_2 enhances the current density in Sn-Pb PSCs by minimizing the reflection, and allowing more light to reach the absorber layer, thereby improving electron-hole pair generation and hence PSC performance.

In **Figure S4**, the 70 nm thickness of MgF_2 appears to balance the reflection reduction and interference effects. At an optimized thickness (70 nm), the ARC reduces reflection over a wide range of wavelengths. When the ARC is too thick (100 nm), interference consequences emerge. Interference can cause destructive interference at specific wavelengths, which reduces overall light absorption.

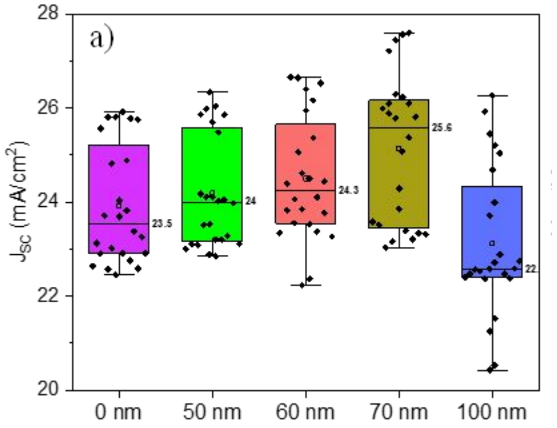

Figure S4. Influence of antireflective coating thicknesses (0 nm, 50 nm, 60 nm, 70 nm, and 100 nm) on J_{SC} of NBG PSCs.

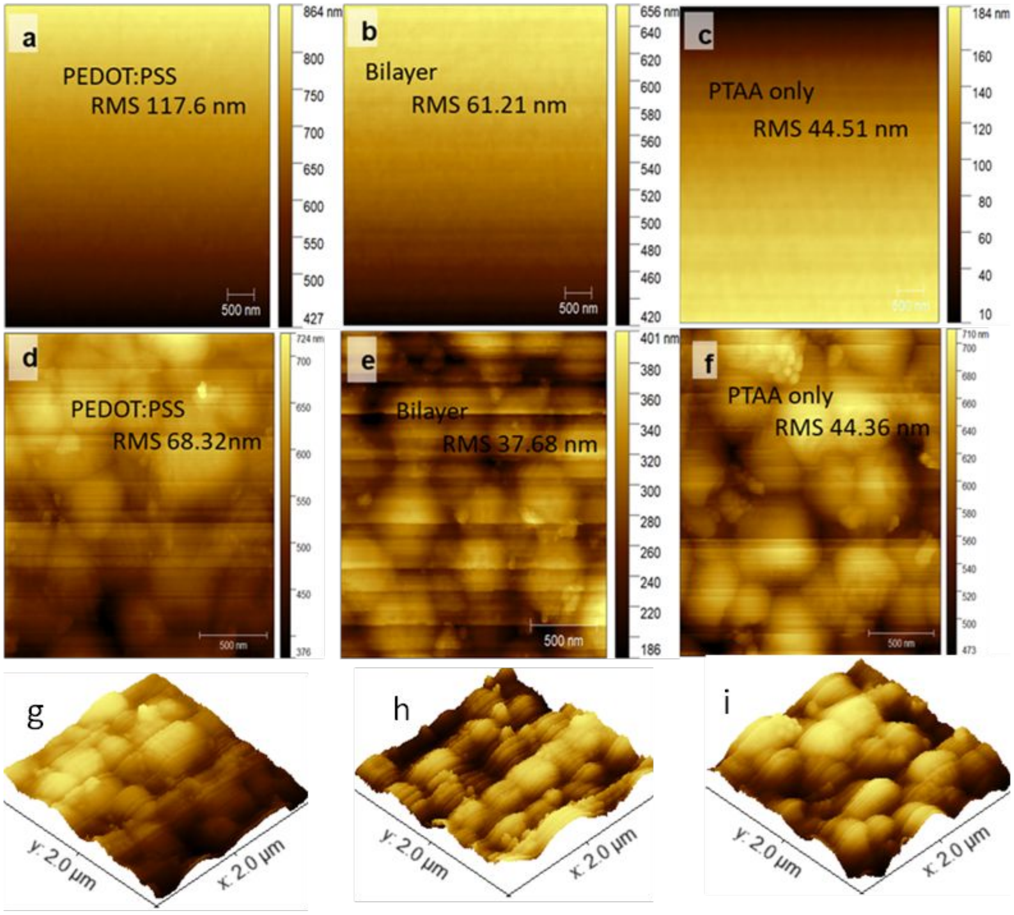

Figure S5. (a-c) AFM images of the three HTLs deposited on ITO (a) ITO/PEDOT:PSS, (b) ITO/PEDOT:PSS/PTAA and (c) ITO/PTAA. (d-f) AFM images of corresponding Sn-Pb perovskite film deposited on the respective HTLs. (g-i)3-D graphs of respective Sn-Pb perovskite film on representative HTLs.

The AFM images in **Figure S5** represent the surface topography of the respective materials, with height variations measured at the nanoscale. From Figure S6 (a-c), the highest value (117.6 nm) implies significant roughness, while the subsequent HTLs (bilayer and PTAA only) values decrease, indicating smoother surfaces. The RMS roughness values for the perovskite layer Figure S6 (d-f) (68.32 nm, 37.68 nm, and 44.36 nm) provide insights into its surface quality, the smaller RMS values suggest smoother surfaces with fewer irregularities. The three-dimensional AFM images of the three HTLs based perovskite are illustrated under Figure S5 (g-i).

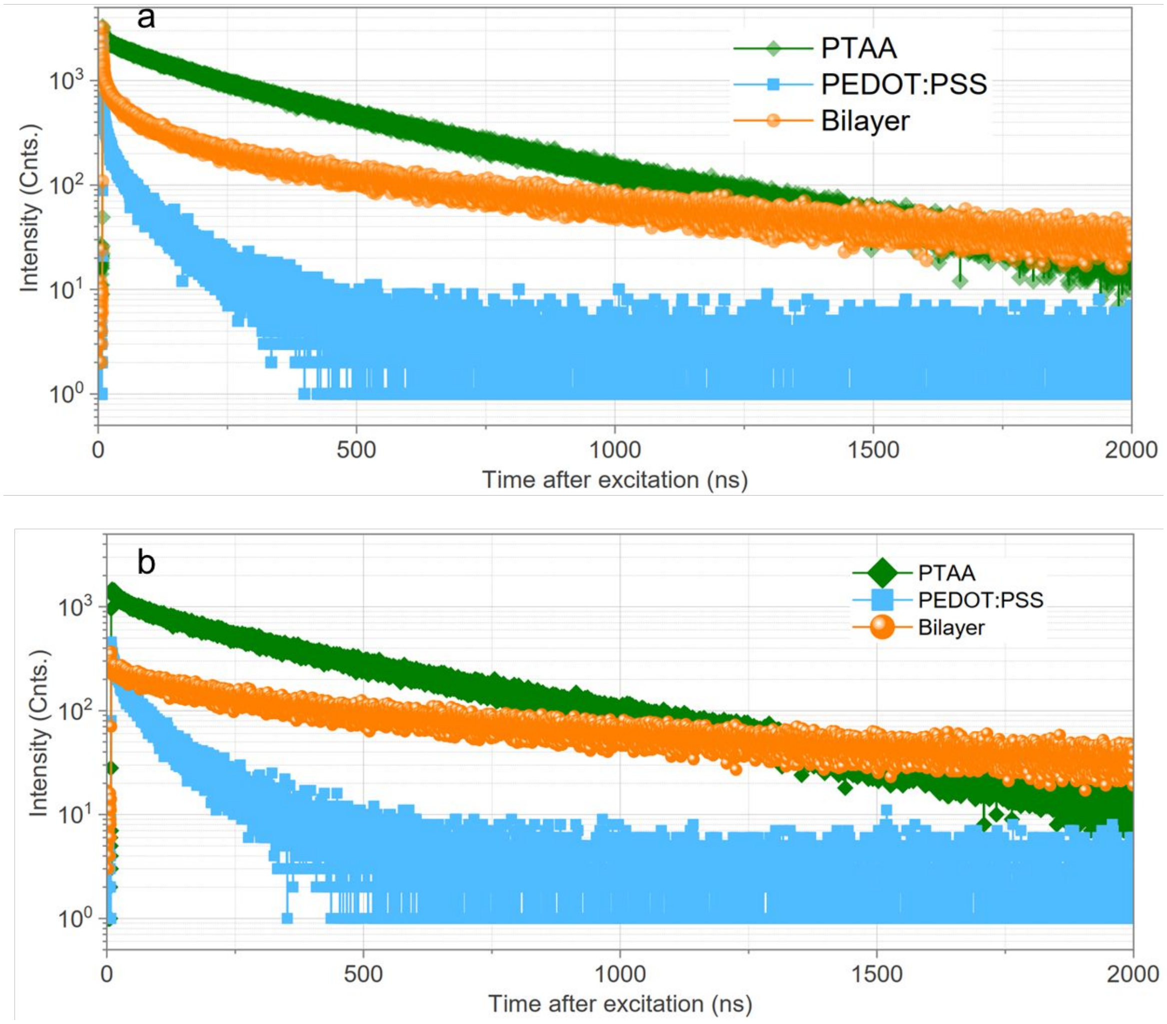

Figure S6. (a) and (b) PL transients of the ITO/HTLs/perovskite excited from glass side and film side, respectively.

Table S1. Photovoltaic parameters of PEDOT:PSS, Bilayer, and PTAA-only based single junction NBG perovskite champion PSCs.

HTLs	Scan directions	J_{SC} (mA/cm^2)	V_{OC} (V)	FF (%)	PCE (%)
PEDOT:PSS	Forward	30.2	0.73	71.2	15.7
	Reverse	30.3	0.80	73.5	17.8
Bilayer	Forward	32.2	0.81	77.0	19.8
	Reverse	32.1	0.82	81.0	20.3
PTAA only	Forward	30.4	0.75	60.0	15.7
	Reverse	30.0	0.77	69.6	16.0

Table S2. Fitting parameters of the time resolved photoluminescence datasets. Four sample sets were analyzed, each consisting of a PEDOT:PSS only sample, a bilayer sample and a PTAA only sample. NBG perovskite was deposited on top of all samples in an identical way. Samples in film side 1 and 2 are illuminated from film sides whereas in glass side 1 and 2 are illuminated from glass side.

\	HTL	τ_1 (ns)	A_1	τ_2 (ns)	A_2	τ_{ave} (ns)
Film side 1	PEDOT:PSS only	87.8	0.283	6.4	1.390	66.4
	Bilayer	339	1.112	76.4	2.129	260.3
	PTAA only	1141	0.226	224.1	0.286	958.2
Glass side 1	PEDOT:PSS only	133.2	0.100	29.8	0.2094	100.3
	Bilayer	485.6	0.708	126.7	0.534	426.6
	PTAA only	1534	0.097	220.1	0.129	1332.2
Film side 2	PEDOT:PSS only	108.4	0.132	11.0	0.461	82.9
	Bilayer	393.5	1.514	111.2	0.953	350.8
	PTAA only	891.0	0.187	83.2	0.649	693.1
Glass side 2	PEDOT:PSS only	155.4	0.127	44.4	0.270	113.4
	Bilayer	533.0	0.591	177.2	0.502	454.7
	PTAA only	1800.0	0.089	253.0	0.126	1540
